# INCREMENTAL HEALTH CARE COSTS OF A SIMPLE MEASURE OF PHENOTYPIC FRAILTY IN OLDER ADULTS

**DOI:** 10.1093/geroni/igae098.1772

**Published:** 2024-12-31

**Authors:** Kristine Ensrud, John Schousboe, Howard Fink, Allyson Kats, Kerry Sheets, Brent Taylor, Cynthia Boyd, Lisa Langsetmo

**Affiliations:** University of Minnesota, Minneapolis, Minnesota, United States; HealthPartners, St. Paul, Minnesota, United States; Minneapolis Veterans Affairs Health Care System, Minneapolis, Minnesota, United States; University of Minnesota, Minneapolis, Minnesota, United States; Hennepin Healthcare, Minneapolis, Minnesota, United States; Minneapolis Veterans Affairs Health Care System, Minneapolis, Minnesota, United States; Johns Hopkins University, Baltimore, Maryland, United States; Minneapolis Veterans Affairs Health Care System, Minneapolis, Minnesota, United States

## Abstract

Frailty defined by the CHS phenotype is associated with higher subsequent total healthcare expenditures in community-dwelling Medicare beneficiaries after accounting for claims-based cost indicators. However, frailty assessment using the 5 component CHS phenotype is often not feasible in clinical practice. Our objective was to determine whether frailty assessed using the simple SOF phenotype (3 components: weight loss, inability to rise from chair 5 times without using arms, poor energy) is associated with subsequent incremental costs after accounting for claims-based measures of multimorbidity and frailty. We utilized a multi-cohort dataset with frailty phenotype measurements linked with Medicare claims composed of 8157 community-dwelling fee-for-service beneficiaries. Healthcare costs were ascertained for 36 months after frailty assessment. Average annualized total healthcare costs (2023 US dollars) were $14945 in women and $15693 in men. After accounting for claims-based indicators of multimorbidity and frailty, average incremental costs of SOF phenotypic frailty (2 or 3 components) vs robust (none) were $7088 in women and $5909 in men, only slightly lower than incremental costs of CHS phenotypic frailty ($9372 in women and $6518 in men). SOF phenotypic frailty in both sexes was associated with higher subsequent expenditures in inpatient, skilled nursing facility and home healthcare sectors. In conclusion, frailty assessed by the parsimonious SOF phenotype is associated with higher subsequent total and sector-specific expenditures after accounting for claims-based indicators. SOF phenotypic frailty can be readily assessed in space-constrained clinic exam rooms and time-limited clinical practice settings to improve identification of older adults at high risk of costly care.

